# A MicroRNA Network Controls *Legionella pneumophila* Replication in Human Macrophages via LGALS8 and MX1

**DOI:** 10.1128/mBio.03155-19

**Published:** 2020-03-24

**Authors:** Christina E. Herkt, Brian E. Caffrey, Kristin Surmann, Sascha Blankenburg, Manuela Gesell Salazar, Anna L. Jung, Stefanie M. Herbel, Kerstin Hoffmann, Leon N. Schulte, Wei Chen, Alexandra Sittka-Stark, Uwe Völker, Martin Vingron, Annalisa Marsico, Wilhelm Bertrams, Bernd Schmeck

**Affiliations:** a Institute for Lung Research, Universities of Giessen and Marburg Lung Center, Philipps University Marburg, Marburg, Germany; b Computational Molecular Biology, Max Planck Institute for Molecular Genetics, Berlin, Germany; c Department of Functional Genomics, University Medicine Greifswald, Greifswald, Germany; d Institute for Lung Research/iLung, Research Group RNA-Biology of Inflammation and Infection, Philipps University, Marburg, Germany; e Department of Biology, Southern University of Science and Technology, Shenzhen, Guangdong, China; f Institute of Computational Biology, Helmholtz Zentrum Muenchen, Neuherberg, Germany; g Department of Medicine, Pulmonary and Critical Care Medicine, University Medical Center Giessen and Marburg, Philipps University, Marburg, Germany; h Center for Synthetic Microbiology (SYNMIKRO), Philipps University Marburg, Marburg, Germany; i German Center for Infection Research (DZIF), partner site Giessen-Marburg-Langen, Marburg, Germany; University of Michigan—Ann Arbor

**Keywords:** miRNA, infection, macrophage, MX1, bacteria, galectin-8, *Legionella pneumophila*, TP53, RIG-I, DDX58, infectious disease, inflammation, macrophages, microRNA

## Abstract

Legionella pneumophila is an important cause of pneumonia. It invades alveolar macrophages and manipulates the immune response by interfering with signaling pathways and gene transcription to support its own replication. MicroRNAs (miRNAs) are critical posttranscriptional regulators of gene expression and are involved in defense against bacterial infections. Several pathogens have been shown to exploit the host miRNA machinery to their advantage. We therefore hypothesize that macrophage miRNAs exert positive or negative control over *Legionella* intracellular replication. We found significant regulation of 85 miRNAs in human macrophages upon L. pneumophila infection. Chromatin immunoprecipitation and sequencing revealed concordant changes of histone acetylation at the putative promoters. Interestingly, a trio of miRNAs (miR-125b, miR-221, and miR-579) was found to significantly affect intracellular L. pneumophila replication in a cooperative manner. Using proteome-analysis, we pinpointed this effect to a concerted downregulation of galectin-8 (LGALS8), DExD/H-box helicase 58 (DDX58), tumor protein P53 (TP53), and then MX dynamin-like GTPase 1 (MX1) by the three miRNAs. In summary, our results demonstrate a new miRNA-controlled immune network restricting *Legionella* replication in human macrophages.

## Abstract (precis)

Cases of Legionella pneumophila pneumonia occur worldwide, with potentially fatal outcome. When causing human disease, *Legionella* injects a plethora of virulence factors to reprogram macrophages to circumvent immune defense and create a replication niche. By analyzing *Legionella*-induced changes in miRNA expression and genomewide chromatin modifications in primary human macrophages, we identified a cell-autonomous immune network restricting *Legionella* growth. This network comprises three miRNAs governing expression of the cytosolic RNA receptor DDX58/RIG-I, the tumor suppressor TP53, the antibacterial effector LGALS8, and MX1, which has been described as an antiviral factor. Our findings for the first time link TP53, LGALS8, DDX58, and MX1 in one miRNA-regulated network and integrate them into a functional node in the defense against L. pneumophila.

## INTRODUCTION

Legionella pneumophila is a causative agent of pneumonia, which is typically characterized as an acute respiratory infection with potentially lethal outcome. In 2016, pneumonia was the most deadly communicable disease worldwide, accounting for 3 million fatalities (1). L. pneumophila is a Gram-negative, rod-shaped, and aerobic bacterium with a single polar flagellum (2). Humans are accidental hosts for *Legionella*, and exposure causes Pontiac fever or the more severe Legionnaires’ disease. The general route of infection in humans is the inhalation of contaminated aerosols (3, 4). After transmission to the lung, bacteria invade alveolar macrophages and replicate (5, 6). Utilizing the Dot/Icm type IV secretion system (T4SS), which is essential for the pathogenesis of *Legionella* (7), more than 330 effector proteins are secreted into the host cell cytosol and repurpose the host cell machinery to ensure *Legionella* survival. The transformation of the phagosome into a *Legionella*-containing vacuole (LCV) is a crucial step, since it enables safe and efficient bacterial replication within the host cells (8). This host subversion is an essential feature of *Legionella* pathogenesis. Although some bacterial factors actively inhibit translation of host mRNA (9, 10), another level of gene-specific reprogramming that is potentially accessible to *Legionella* is represented by miRNA-mediated posttranscriptional regulation.

miRNAs are endogenous, noncoding, single-stranded RNA molecules with a length of 20 to 24 nucleotides that regulate gene expression at the posttranscriptional level (11). Regulation by miRNAs has been studied in a wide range of leukocytes and in the immune response of nonleukocytes. Indeed, the activation of innate immune cells, including monocytes, macrophages, dendritic cells, and natural killer cells, is partly controlled by miRNAs (12–15).

The microRNAs miR-9, miR-127, miR-155, and miR-125b have been shown to be involved proinflammatory macrophage activation, whereas miR-124, miR-223, miR-34a, let-7c, miR-132, miR-146a, and miR-125a-5p promote the induction of alternative macrophage activation by targeting various transcription factors and adaptor proteins (16, 17). Furthermore, miR-146, miR-155, and let-7 miRNAs act in concert to balance phagocyte immune signaling and cytokine production in response to bacterial infection through feed-forward and feed-back mechanisms (18, 19). Intriguingly, several pathogens seem to have evolved strategies to exploit host miRNA function to their own advantage (20, 21). Furthermore, individual miRNAs have been shown to impact on intracellular bacterial replication (22).

We therefore inferred that miRNAs are implicated in the interplay of intracellular *Legionella* and macrophages of the human host. To address this hypothesis, we performed time course RNA sequencing analysis of the small RNA fraction of control- and *Legionella*-treated macrophages to determine the infection-associated miRNA expression changes. We show that L. pneumophila infection of human macrophages leads to altered expression of numerous miRNAs, which we could partly attribute to acetylation changes at their primary transcript promoters. We identified the host miRNAs miR-125b, miR-221, and miR-579 as decisive factors for intracellular bacterial replication, and we pinpointed this effect to the downregulation of MX dynamin-like GTPase 1 (MX1). While not a direct miRNA target itself, we found downregulation of MX1 to be mediated via targeting of tumor protein P53 (TP53) by miR-125b and DExD/H-box helicase 58 (DDX58) by miR-221. MX1 has already been described to possess antiviral properties (23–25). Here, we establish that MX1 downregulation enhances L. pneumophila replication. A third factor, galectin-8 (LGALS8), which is also known to limit bacterial replication (26, 27), was targeted by miR-579. In summary, MX1 and LGALS8 were identified as effector molecules downstream of regulatory events mediated by three miRNAs (miR-125b, miR-221, and miR-579) restricting L. pneumophila replication within human macrophages.

(Part of this work is included in the doctoral thesis of C. E. Herkt at Philipps University Marburg, Marburg, Germany, in 2018.)

## RESULTS

### miRNAs are differentially expressed in blood-derived macrophages after *L. pneumophila* infection and alter bacterial replication.

**FIG 1:**
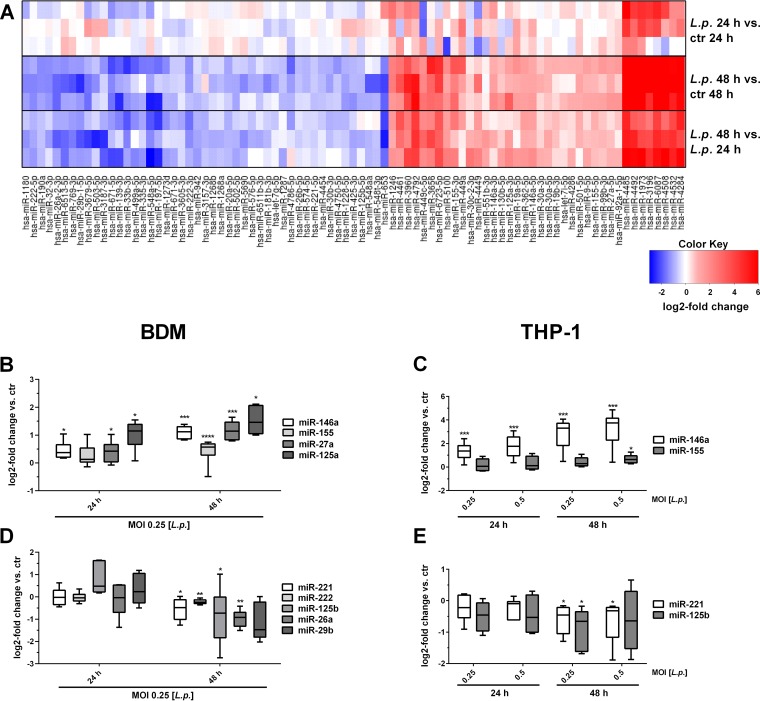
Differentially expressed miRNAs in blood-derived macrophages after L. pneumophila infection. BDMs from three different donors were infected with L. pneumophila at an MOI of 0.25 or left untreated as a control (ctr). Samples were taken 24 and 48 h postinfection. RNA was isolated and used for library preparation and enriched for small RNAs using a TruSeq small RNA kit. Sequencing was performed in a multiplexed run of 1 × 51 cycle +7 (index). (A) Heatmap of the miRNA log_2_ fold change over the respective controls. The expression of distinct upregulated (B and C) and downregulated (D and E) miRNAs in BDMs and THP-1 cells after L. pneumophila infection was validated by qPCR using TaqMan assays and are displayed as log_2_ fold changes. Boxes show the upper and lower quartiles with medians and whiskers indicating minimal and maximal values of five to nine independent biological replicates for dysregulated miRNAs. Paired *t* tests were performed. *, *P* ≤ 0.05; **, *P* ≤ 0.01; ***, *P* ≤ .001; ****, *P* ≤ 0.0001 (compared to the corresponding control).

In order to characterize the miRNA-mediated regulatory network upon *Legionella* infection, human blood-derived macrophages (BDMs) were infected with L. pneumophila at a multiplicity of infection (MOI) of 0.25 for 24 and 48 h, and miRNA expression changes were determined by means of small RNA sequencing with respect to control samples collected at both time points (deposited under GEO accession number GSE125559). Comparative analyses revealed that the expression of 85 miRNAs changed significantly as a function of infection and time (Fig. 1A). Most miRNAs were found to be differentially regulated 48 h after treatment in comparison to both control and 24-h expression values, while only four miRNAs were found to be significantly deregulated after 24 h compared to control (see Table S1 in the supplemental material). Among the 38 upregulated miRNAs, miR-146a and miR-155 were identified, which are known to play a crucial role in the inflammatory TLR response after bacterial infection (28–30). Further upregulated miRNAs that have not yet been characterized in the context of infection include, e.g., miR-3196 or miR-4284. Of the 47 downregulated miRNAs, miR-653, miR-548a, and miR-671 displayed the highest fold changes, but since no described function in the immune response for these miRNAs are described in the literature, they were not further analyzed.

10.1128/mBio.03155-19.9 TABLE S1 Significantly regulated microRNAs as determined by sequencing. MicroRNAs that were differentially expressed at 24 h or 48 h post infection in the indicated comparisons are shown with their fold change, *P* value, and adjusted *P* value (Benjamini-Hochberg corrected for multiple testing). Download Table S1, XLSX file, 0.03 MB. Copyright © 2020 Herkt et al. 2020 Herkt et al. This content is distributed under the terms of the Creative Commons Attribution 4.0 International license.

Among the significantly downregulated miRNAs miR-221 and miR-125b were of particular interest, since they have been implicated in posttranscriptional control of monocytic immune tolerance to bacterial components (31). These miRNAs were validated by quantitative PCR (qPCR) in BDMs from six independent donors (Fig. 1B and D) and in the THP-1 cell line (Fig. 1C and E), which yielded comparable results. Additional testing of cytokine secretion and gene expression (see Fig. S2 in the supplemental material) supported the notion of THP-1 cells as a suitable model for BDMs in further experiments to study L. pneumophila-regulated miRNAs in macrophages.

**FIG 2:**
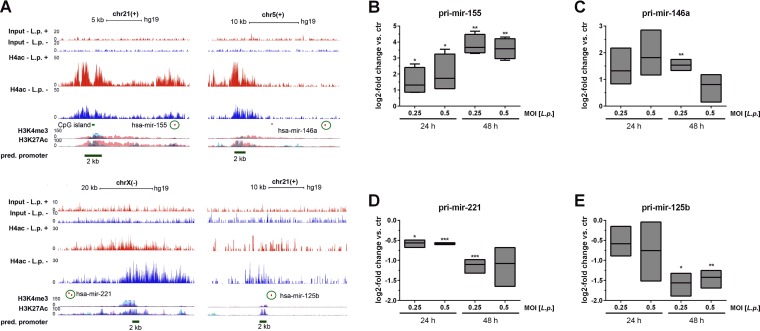
Infection-related chromatin changes on miRNA promoters. The alterations of the acetylation pattern at the miRNA promoter regions of miR-155, miR-146a, miR-221, and miR-125b after L. pneumophila infection in BDMs were investigated. BDMs were infected with L. pneumophila for 1 h, and ChIP was performed using a pan-ac-H4-antibody (based on data published by Du Bois et al. [32]). (A) After sequencing, the enrichment of H4 acetylation at the miR promoters was analyzed. As confirmation for active sites in the promoter region, IP data for H3K4me3 and H3K27Ac provided by the Encode UCSC browser are shown. Horizontal dark green lines indicate the genomic locations of the promoter regions (2,000 bp) for the four analyzed miRNAs corresponding to given coordinates. Circles highlight the mature transcript of each miRNA. The expression of the pri-miRs (B, C, D, and E) in BDMs after L. pneumophila infection (MOI of 0.25 or 0.5) was examined by qPCR and is displayed as log_2_ fold changes. Boxes show the upper and lower quartiles, with medians (when *n* = 3 independent biological replicates) and whiskers indicating minimal and maximal values (when *n* = 4 independent biological replicates). Paired *t* tests were performed. *, *P* ≤ 0.05; **, *P* ≤ 0.01; ***, *P* ≤ 0.001 (compared to the corresponding control).

The promoter regions of miR-155 and miR-146a (upregulated) and of miR-221 and miR-125b (downregulated) were investigated using our own previously published H4 acetylation chromatin immunoprecipitation (ChIP) sequencing data (GEO accession number GSE80214) from *Legionella*-infected BDMs (32). The promoter regions of miR-155 and miR-146a revealed an increased H4 acetylation upon L. pneumophila infection at an MOI of 10 for 1 h, with linear fold changes of 1.9 and 1.3, respectively, while miR-221 and miR-125b, mainly the paralog hsa-miR-125b-2 on chromosome 21, showed a decreased H4 acetylation at their promoters with linear fold changes of 0.77 and 0.54, respectively (Fig. 2A).

As these results suggested transcriptional regulation of miRNA expression in L. pneumophila infection, as opposed to changes in miRNA processing, the pri-miRNA expression levels of miR-146a, miR-155, miR-125b, and miR-221 were analyzed by qPCR. pri-mir-16 served as an endogenous control (33, 34). Upregulation of pri-mir-155 and -146a (Fig. 2B and C), as well as downregulation of pri-miR-221 and -125b (Fig. 2D and E), was observed after infection with L. pneumophila, which led us to conclude that regulation of these miRNAs upon infection occurs at the transcriptional level. In summary, our miRNA profiling data show, among several key immune-associated miRNAs, the downregulation of immune-tolerance-associated miRNAs miR-221 and miR-125b during *Legionella* infection.

### Immune-responsive miRNAs facilitate intracellular *Legionella* replication.

**FIG 3:**
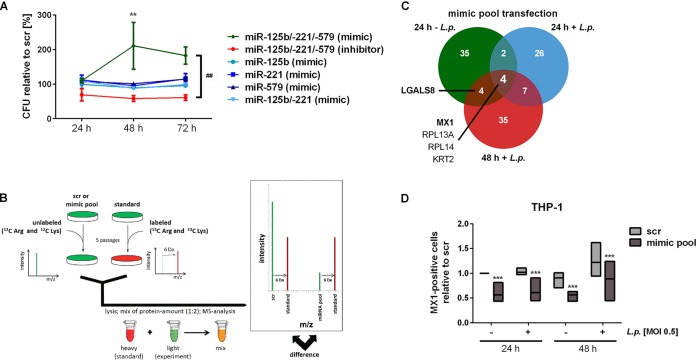
Influence of miRNAs on bacterial replication in macrophages through downregulation of MX1 protein. THP-1 cells were transfected with miR-125b/-221/-579 mimics or inhibitors plus scrambled control (scr) at a final concentration of 30 nM. At 24 h posttransfection, THP-1 cells were activated with PMA for another 24 h and then infected with L. pneumophila at an MOI of 0.5. (A) Bacterial replication was determined by a CFU assay at the indicated time points. The bacterial yield is depicted as the percent relative to CFU count of scr-transfected cells at every time point, and values are shown as means ± the standard errors of the mean (SEM) of three to four independent biological replicates. (B) SILAC-supported quantitative proteomics was used to analyze the proteome of THP-1 cells treated as in panel A. (C) The numbers of proteins that were downregulated after simultaneous precursor transfection of all three miRNAs compared to scramble transfection are shown. (D) The relative numbers of MX1-positive cells upon miRNA transfection were analyzed by flow cytometry. Boxes show the upper and lower quartiles with median of three independent biological replicates. Significance was assessed by two-way ANOVA with Tukey’s (for CFU assay) or Sidak’s (for MX1 staining) correction. *, *P* ≤ 0.05; **, *P* ≤ 0.01; ***, *P* ≤ 0.001 (compared to the corresponding control). ##, *P* ≤ 0.01 (for global treatment effect).

Previously, miR-221 and miR-125b, along with a third miRNA, miR-579, have been reported to confer immune tolerance of monocytic cells to bacterial components (31, 35). We therefore hypothesized that the observed downregulation of this miRNA pool (see Fig. S1 in the supplemental material) might be involved in promoting antimicrobial defense. In a pilot set of experiments, the impact of miR-125b, miR-221, and miR-579 on the intracellular replication of L. pneumophila was determined. To this end, THP-1 cells were transfected with a pool of synthetic miR-125b, miR-221, and miR-579 precursors or inhibitors and were subsequently infected. Intracellular replication of L. pneumophila was monitored at 24, 48, and 72 h. Although treatment with the miRNA inhibitor pool resulted in reduced intracellular replication compared to a scramble control (scr), transfection of all three miRNA precursors resulted in a strong increase of intracellular replication. Individual transfection of a single miRNA precursor/inhibitor did not change replication of L. pneumophila (Fig. 3A). To further test assay validity, we verified *Legionella* replication in THP-1 cells with concomitant scramble RNA transfection for up to 72 h (Fig. S3). These observations point to a concerted action of all three miRNAs in pathogen replication control in macrophages.

10.1128/mBio.03155-19.2 FIG S1 miRNAs miR-579, miR-221, and miR-125b are downregulated upon infection of BDMs with Legionella pneumophila. Log_2_-fold changes were calculated based on the read counts from the small RNA sequencing experiment. While read counts for miR-579 were very low (<5 reads per sample), they followed the consistent pattern of downregulation observed across all three miRNAs. Download FIG S1, TIF file, 0.05 MB. Copyright © 2020 Herkt et al. 2020 Herkt et al. This content is distributed under the terms of the Creative Commons Attribution 4.0 International license.

10.1128/mBio.03155-19.3 FIG S2 Proinflammatory cytokine release of macrophages upon infection with L. pneumophila BDMs or PMA-differentiated THP-1 cells were infected with L. pneumophila at an MOI of 0.25 or 0.5 for 24 or 48 h, respectively. Secretion of IL-1β (A and B), IL-6 (C and D), GM-CSF (E and F), TNF-α (G and H) and IL-10 (I and J) was measured using a multiplex Luminex assay. Boxes show the upper and lower quartiles with medians of three independent biological replicates. Paired *t* tests were performed: *, *P* ≤ 0.05; **, *P* ≤ 0.01; **, *P* ≤ 0.001 compared to the corresponding control. Download FIG S2, TIF file, 0.1 MB. Copyright © 2020 Herkt et al. 2020 Herkt et al. This content is distributed under the terms of the Creative Commons Attribution 4.0 International license.

10.1128/mBio.03155-19.4 FIG S3 Replication of Legionella pneumophila in THP-1 cells. PMA differentiated THP-1 cells were transfected with scramble control RNA and infected with L. pneumophila at an MOI of 0.5, and replication was monitored by CFU assay for up to 72 h. Absolute CFU/ml are shown as means ± the SEM of 11 independent experiments. Download FIG S3, TIF file, 0.03 MB. Copyright © 2020 Herkt et al. 2020 Herkt et al. This content is distributed under the terms of the Creative Commons Attribution 4.0 International license.

### MX1 is involved in miRNA-dependent control of *Legionella* replication.

To address the underlying mechanism, THP-1 cells were transfected with the miRNA precursor pool and additionally infected with L. pneumophila at an MOI of 0.5 for 24 and 48 h or left untreated as a control. As posttranscriptional regulation of coding genes commonly must also be evident on the protein level to be functional, the proteome patterns of these samples were comparatively profiled by mass spectrometry (MS) (Fig. 3B). This method comparatively profiles differences in protein levels based on isotope-labeled amino acid incorporation. Quantitative data were obtained for 1,670 proteins; of these, 113 proteins displayed reductions in protein level in response to transfection with the miRNA precursor pool in comparison to scr-transfected cells with (24 h + L. pneumophila and 48 h *+* L. pneumophila) or without infection (24 h – L. pneumophila) by L. pneumophila. The impact of the transfection with the miRNA precursor pool or in combination with L. pneumophila infection is illustrated in Fig. 3C with regard to these 113 proteins. Of note, miRNA precursor transfection alone or in combination with L. pneumophila infection triggered reductions in the level of MX dynamin-like GTPase 1 (MX1), as well as RPL13A, RPL14, and KRT2, in comparison to the respective controls. In addition, the levels of LGALS8 (galectin-8) were reduced following miRNA precursor pool transfection in cells infected with L. pneumophila for 48 h and in uninfected cells. To elucidate the miRNA-mediated influence on L. pneumophila replication, the focus was turned to MX1 and LGALS8, given their documented role in infection control.

**FIG 4:**
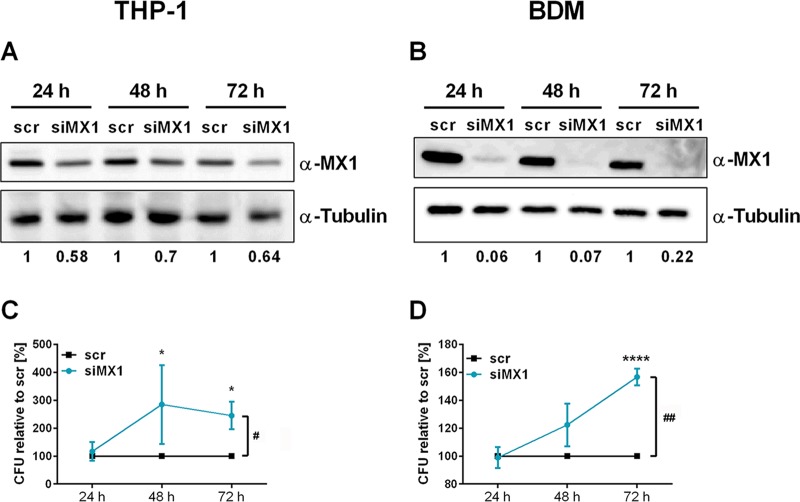
Downregulation of MX1 enhances L. pneumophila replication in macrophages. THP-1 cells or BDMs were transfected with an siRNA-pool targeting MX1 (siMX1) or with scrambled siRNA as a control (scr). At 24 h posttransfection, THP-1 cells were stimulated with PMA (20 nM) for another 24 h. Differentiated THP-1 cells or BDMs were infected as shown. Knockdown of MX1 protein in THP-1 cells (A) and BDMs (B) was verified and quantified by Western blotting. CFU were determined 24, 48, and 72 h postinfection after MX1 knockdown in THP-1 cells (C) or BDMs (D). Mean values ± the SEM of three to seven independent biological experiments are depicted. Significance was assessed by two-way ANOVA with Sidak**’**s correction. *, *P* ≤ 0.05; **, *P* ≤ 0.01; ***, *P* ≤ 0.001 (compared to the corresponding control). #, *P* ≤ 0.05, ##, *P* ≤ 0.01 (for global treatment effects).

To validate the results of the proteome profiling, intracellular MX1 was investigated by flow cytometry. After transfection with the miRNA precursor pool, we observed a reduced MX1 signal under all tested conditions (Fig. 3D; see also the exemplary histogram in Fig. S4). Of note, mRNA levels of MX1 remained unaffected. In order to evaluate a potential direct effect of MX1 on infection, THP-1 cells or BDMs were transfected with an siRNA-pool targeting MX1 (siMX1). Subsequently, cells were infected with L. pneumophila at an MOI of 0.5. Knockdown of MX1 protein was confirmed by Western blot analysis and densitometric quantification in THP-1 cells (Fig. 4A) and BDMs (Fig. 4B). In THP-1 cells, siRNAs reduced MX1 protein levels to approximately 64% over 72 h. Importantly, knockdown of MX1 resulted in increased L. pneumophila replication in THP-1 cells (Fig. 4C) and BDMs (Fig. 4D), which was similar to the effect upon miRNA precursor transfection (Fig. 3A).

10.1128/mBio.03155-19.5 FIG S4 MX1 downregulation following precursor transfection of the miRNA-pool. THP-1 cells were transfected with miRNA-125b, miR-221, and miR-579 miRNA precursors (miRNA mimic pool) at a final concentration of 30 nM. For control, a scrambled precursor (scr) was transfected. Twenty-four h post transfection, THP-1 cells were differentiated with 20 nM PMA for 24 h and infected with L. pneumophila. at an MOI of 0.5 for 24 and 48 h or left untreated as control. Expression of MX1 was examined by qPCR and displayed as log_2_-fold change. Boxes show the upper and lower quartiles with median and whiskers indicate minimal and maximal values of four independent biological replicates (A). For analysis of the intracellular protein expression of MX1, an indirect fluorescence staining followed by cytometric analysis was performed. Transfected cells were fixed with 4% PFA and stained with an anti-MX1 antibody or an unspecific IgG antibody as control. Finally, cells were stained by a secondary antibody labelled with Alexa Fluor 555. The percentages of MX1-positive cells were determined with FlowJo 7.6.5. One representative histogram is shown in panel B. Download FIG S4, TIF file, 0.1 MB. Copyright © 2020 Herkt et al. 2020 Herkt et al. This content is distributed under the terms of the Creative Commons Attribution 4.0 International license.

**FIG 5:**
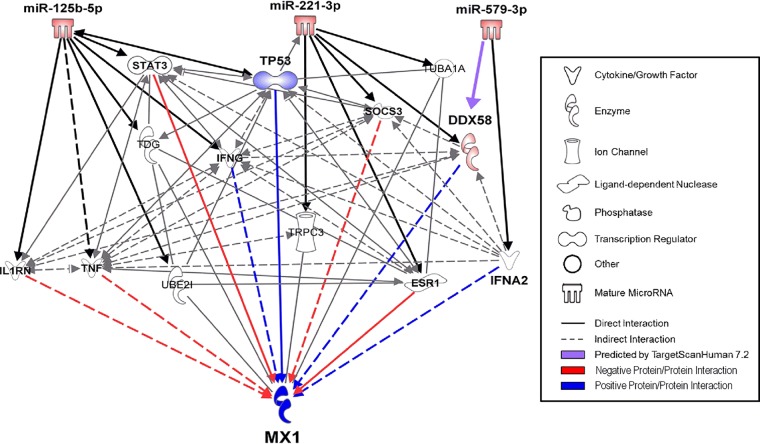
MX1 is not directly targeted by the miRNA-pool. IPA was performed to investigate the connection between the miRNA pool (miR-125b, miR-221, and miR-579) and the MX1 protein. Filters were set to only include experimentally observed or high-confidence-predicted miRNA-mRNA interaction partners. Output was limited to 13 total candidates. The newly found miRNA targets were interconnected using all available data sources in IPA with restriction to experimentally validated or high-confidence-predicted interactions. Direct or indirect relationships between molecules are indicated by solid or dashed lines, respectively. Blue lines depict positive regulations, while red lines indicate negative regulations. Molecule classes (cytokine, enzyme, ion channel, ligand-dependent nuclease, phosphatase, transcription factor, other, or mature miRNAs) are indicated by distinct symbols.

**FIG 6:**
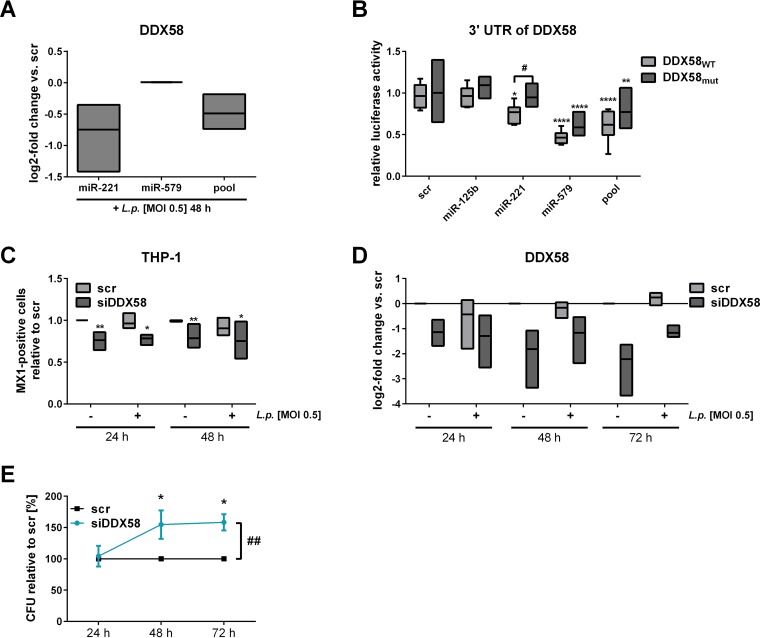
DDX58 is targeted by miR-221 and influences bacterial replication. THP-1 cells were transfected either with miR-221 mimic, miR-579 mimic, or the miRNA mimic pool (miR-125b, miR-221, and miR-579) at a final concentration of 30 nM. As a control, a scrambled precursor (scr) was transfected. At 24 h posttransfection, THP-1 cells were activated with PMA for another 24 h and then infected with L. pneumophila at an MOI of 0.5. (A) DDX58 expression was determined via qPCR and is displayed as the log_2_ fold change. Luciferase reporter assay analyses were carried out in HEK-293T cells. The plasmid harbored either the wild-type (DDX58_WT_) or the mutated (DDX58_mut_) version of the 3′ UTR of DDX58. (B) Ratios of *Renilla* and firefly luciferase luminescence were normalized to the vector without insert. (C) Intracellular MX1 upon miRNA pool transfection was quantified by flow cytometric analysis, and the relative numbers of MX1-positive cells were calculated. (D) BDMs were transfected with a small interfering RNA-pool targeting DDX58 (siDDX58) or with a scrambled siRNA as control (scr) and infected at an MOI of 0.5 or left untreated. The downregulation of DDX58 expression with siRNA was verified by qPCR and is displayed as the log_2_ fold change. (E) The CFU of *Legionella* were determined 24, 48, and 72 h postinfection after a knockdown of DDX58. Boxes show the upper and lower quartiles, with medians (when *n* = 3 independent biological replicates) and whiskers indicating minimal and maximal values (when *n* = 4 to 6 independent biological replicates). A two-way ANOVA with Sidak’s correction was performed. *, *P* ≤ 0.05; **, *P* ≤ 0.01; ***, *P* ≤ 0.001 (compared to scramble). #, *P* ≤ 0.05 compared to the wild type (B); ##, *P* ≤ 0.01 (for global treatment effects) (E).

The expected miRNA mode of action prompted us to investigate the 3′ untranslated region (UTR) of MX1 for binding sites of the miRNA pool (miR-125b, miR-221, and miR-579). *In silico* analysis by TargetScan Human 7.1 revealed no significant binding sites for any miRNA. Manual search for seed-compatible regions in the 3′ UTR was equally unsuccessful. Qiagen Ingenuity Pathway Analysis (IPA) yielded a complex network of predicted and published targets of each miRNA. Filters were set to only include experimentally observed or high-confidence predicted miRNA/mRNA interaction partners. The newly found miRNA targets were interconnected using all available data sources in IPA with restriction to experimentally validated or high-confidence predicted interactions in humans (Fig. 5). Positive regulators of MX1 (blue) were prioritized over negative regulators (red), since miRNAs usually downregulate their targets. This filter strategy left 4 of 13 possible candidates for MX1 regulation mediated by the miRNA pool, two of which (gamma interferon [IFNG] and interferon α2 [IFNA2]) were discarded due to very low expression in THP-1 cells. The two remaining candidates were DExD/H-box helicase 58 (DDX58), also known as RIG-I, and tumor protein P53 (TP53). Intriguingly, the nucleic acid sensor DDX58 is a known regulator of host defense against *Legionella* infection (36). *In silico* analysis (TargetScan Human 7.1) of the DDX58 3′ UTR revealed one binding site for miR-579 and two binding sites for miR-221 (Fig. S5). Transfection of miR-221 alone and of the miRNA precursor pool resulted in decreased DDX58 mRNA expression. In comparison, transfection of miR-579 precursor alone did not result in reduced DDX58 mRNA expression (Fig. 6A). Analysis of miR-221 and miR-579 targeting of the DDX58 3′ UTR (DDX58_WT_) by a luciferase reporter assay revealed significant functional interaction at 72 h after cotransfection of miR-221, miR-579, or the miRNA precursor pool (Fig. 6B). Binding specificity was tested by mutation of the putative miRNA binding sites (DDX58_mut_) and was positively ascertained for miR-221. The specificity of the miR-579 binding to the putative target site could not be confirmed and was hence disregarded.

10.1128/mBio.03155-19.6 FIG S5 miRNA binding sites within the 3′ UTR of the respective targets. (A) There are two potential miRNA binding sites for miR-221 and one for miR-579 in the 3′ UTR of DDX58. (B) The 3′ UTR of TP53 possesses only one miRNA binding site for miR-125b, while the 3′ UTR of LGALS8 has two potential binding sites for the miR-579. Vertical bars represent canonical base pairing (C-G; A-U), while horizontal lines indicate the seed regions of the miRNAs. The mutated bases are depicted in red. Download FIG S5, TIF file, 0.1 MB. Copyright © 2020 Herkt et al. 2020 Herkt et al. This content is distributed under the terms of the Creative Commons Attribution 4.0 International license.

Upon small interfering RNA (siRNA)-mediated DDX58 knockdown, MX1 protein levels were at 75% compared to scramble transfected cells as determined by flow cytometry (Fig. 6C). siDDX58-treated BDMs were infected with L. pneumophila at an MOI of 0.5 to assess replication at 24, 48, and 72 h postinfection. Successful knockdown was verified via qPCR (Fig. 6D) and resulted in an up to 150% replication of L. pneumophila in BDMs (Fig. 6E), which was also shown in differentiated THP-1 cells (Fig. S6).

10.1128/mBio.03155-19.7 FIG S6 Knockdown of DDX58 or LGALS8 in THP-1 cells increases L. pneumophila replication. THP-1 cells were transfected with a small interfering RNA targeting DDX58 (siDDX58) or LGALS8 (siLGALS8). As control a scrambled siRNA (scr) was transfected. At 24 h posttransfection, THP-1 cells were stimulated with PMA (20 nM) for 24 h and infected using L. pneumophila at an MOI of 0.5 or left untreated as control. Downregulation of DDX58 (A) or LGALS8 (C) expression with siRNA was verified by qPCR and displayed as log_2_-fold changes. Boxes show the upper and lower quartiles with median of three independent biological replicates. CFUs of *Legionella* were determined 24, 48, and 72 h postinfection following a knockdown of DDX58 (B) or LGALS8 (D). Mean values + the SEM of four or five independent biological experiments are depicted. For the CFU-assays a two-way ANOVA with Sidak’s correction was performed. *, *P* ≤ 0.05; **, *P* ≤ 0.01; ***, *P* ≤ 0.001 compared to the corresponding control. ###, *P* ≤ 0.001 for global treatment effects. Download FIG S6, TIF file, 0.1 MB. Copyright © 2020 Herkt et al. 2020 Herkt et al. This content is distributed under the terms of the Creative Commons Attribution 4.0 International license.

**FIG 7:**
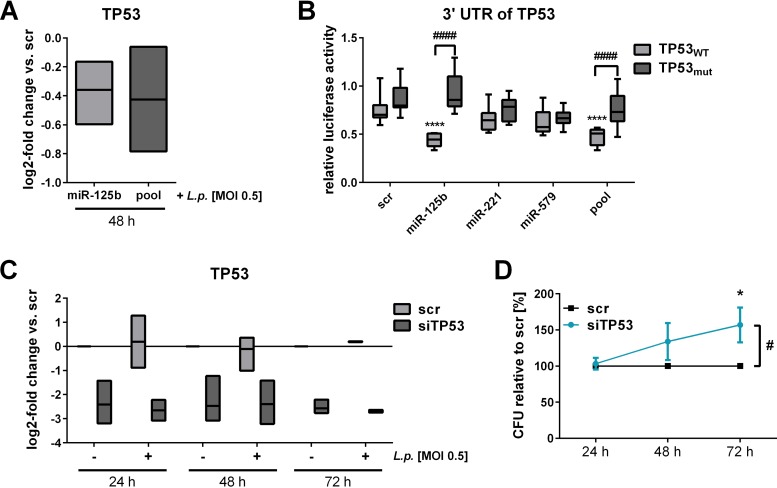
TP53 is targeted by miR-125b and affects L. pneumophila replication. THP-1 cells were transfected with either miRNA-125b mimic or an miRNA mimic pool (miR-125b, miR-221, and miR-579) at a final concentration of 30 nM. As a control, a scrambled precursor (scr) was transfected. At 24 h posttransfection, THP-1 cells were activated with PMA for another 24 h and then infected with L. pneumophila at an MOI of 0.5. (A) TP53 expression was examined via qPCR and is displayed as the log_2_ fold change. Luciferase reporter assay analyses were performed in HEK-293T cells. The plasmid contained either the wild-type (TP53_WT_) or the mutated (TP53_mut_) version of the 3′ UTR of TP53. Ratios of *Renilla* and firefly luciferase luminescence were normalized to the vector without insert. BDMs were transfected with an siRNA pool targeting TP53 (siTP53) or with a scrambled siRNA as a control (scr) and infected at an MOI of 0.5 or left untreated. (C) Downregulation of TP53 expression with siRNA was verified by qPCR and is displayed as the log_2_-fold change. (D) The CFU of *Legionella* were determined 24, 48, and 72 h postinfection after a knockdown of TP53. Boxes show the upper and lower quartiles with median (when *n* = 3 independent biological replicates) and whiskers indicate minimal and maximal values (when *n* = 4 independent biological replicates). A two-way ANOVA with Sidak’s correction was performed. *, *P* ≤ 0.05; **, *P* ≤ 0.01; ***, *P* ≤ 0.001; ****, *P* ≤ 0.0001 (compared to scramble). ####, *P* ≤ 0.0001 (compared to the wild type) (B). #, *P* ≤ 0.05 (for global treatment effects) (D).

TP53, the second candidate to link miRNA precursor transfection to MX1, has previously been validated as a target of miRNA-125b by several studies (37–39). As expected, transfection of miR-125b precursor alone or the miRNA precursor pool reduced TP53 expression in THP-1 cells (Fig. 7A). Accordingly, transfection of miR-125b or the miRNA-pool with the partial 3′ UTR of TP53 (TP53_WT_) containing the predicted miRNA binding sites resulted in a significant decrease of relative luciferase activity compared to scramble transfected cells in a luciferase-based reporter assay (Fig. 7B), which could be reversed by targeted mutation of the putative binding sites (TP53_mut_). CFU assays following efficient siRNA-mediated knockdown of TP53 in BDMs (Fig. 7C) revealed 157% replication at 72 h postinfection compared to the scramble control (Fig. 7D). The effect of TP53 on MX1 has already been described (40).

### LGALS8 is targeted by miRNA-579 and influences *L. pneumophila* replication.

**FIG 8:**
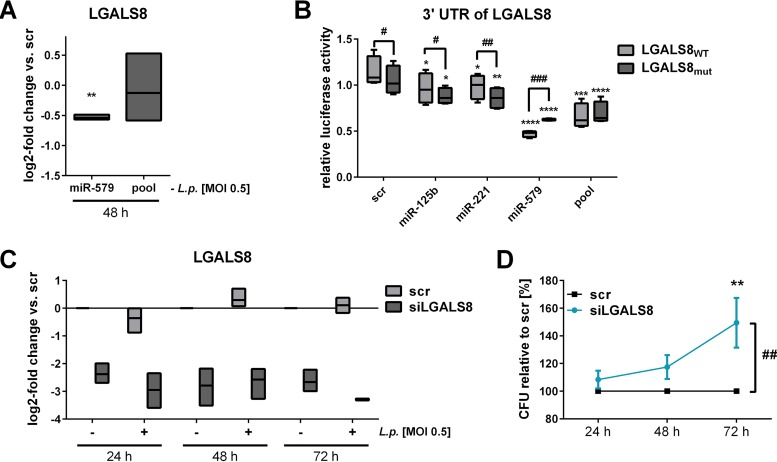
LGALS8 is targeted by miR-579 and influences L. pneumophila replication. THP-1 cells were transfected with either the miR-579 mimic or the miRNA mimic pool (miR-125b, miR-221, and miR-579) at a final concentration of 30 nM. As a control, a scrambled precursor (scr) was transfected. At 24 h posttransfection, THP-1 cells were activated with PMA for another 24 h and then infected with L. pneumophila at an MOI of 0.5. (A) LGALS8 expression was examined with qPCR and is displayed as the log_2_ fold change. Luciferase reporter assay analyses were performed in HEK-293T cells. The plasmid contained either the wild-type (LGALS8_WT_) or the mutated (LAGLS8_mut_) version of the 3′ UTR of LGALS8. (B) Ratios of *Renilla* and firefly luciferase luminescence were normalized to the vector without insert. BDMs were transfected with an siRNA pool targeting LGALS8 (siLGALS8) or with a scrambled siRNA as a control (scr) and infected at an MOI of 0.5 or left untreated as a control. (C) Downregulation of LGALS8 expression with siRNA was verified by qPCR and is displayed as the log_2_-fold change. (D) CFU of *Legionella* were determined 24, 48, and 72 h postinfection after a knockdown of LGALS8. Boxes show the upper and lower quartiles, with medians (when *n* = 3 independent biological replicates) and whiskers indicating minimal and maximal values (when *n* = 4 or 5 independent biological replicates). Two-way ANOVA with Sidak’s correction was performed. *, *P* ≤ 0.05; **, *P* ≤ .01; ***, *P* ≤ 0.001; ****, *P* ≤ 0.0001 (compared to the corresponding control). #, *P* ≤ 0.05; ##, *P* ≤ 0.01; ###*P* ≤ 0.001 (compared to wild type) (B). ##, *P* ≤ 0.01 (for global treatment effects) (D).

As shown, all three miRNAs were necessary to mediate replication of L. pneumophila in macrophages, but our model did not yet include a role for miR-579. Since the protein LGALS8 is known to be involved in antibacterial defense (26, 27, 41) and displayed decreased levels upon miRNA transfection (Fig. 3C), LGALS8 was further investigated. First, the influence of miR-579 on LGALS8 mRNA was assessed. The individual transfection of miR-579 precursor resulted in significantly reduced levels of LGALS8, while transfection with the miRNA pool, which carries reduced per-miRNA concentrations compared to individual transfection, showed no influence (Fig. 8A). Accordingly, we could demonstrate a reduction in LGALS8 protein levels upon miR-579 transfection by flow cytometry (Fig. S7).

10.1128/mBio.03155-19.8 FIG S7 LGALS8 downregulation following miR-579 precursor transfection. THP-1 cells were transfected with miR-579 at a final concentration of 30 nM. For control, a scrambled precursor (scr) was transfected. AT 24 h posttransfection, THP-1 cells were differentiated with 20 nM PMA for 24 h and infected with L. pneumophila at an MOI of 0.5 for 48 h. Intracellular protein expression of LGALS8 was measured by an indirect fluorescence staining followed by cytometric analysis. Transfected cells were fixed with 4% PFA and stained with an anti-LGALS8 antibody or an unspecific IgG antibody as control. Finally, cells were stained by a secondary antibody labelled with Alexa Fluor 555. (A) One representative FACS plot is shown. The percentages of LGALS8-positive cells were determined with FlowJo 7.6.5. (B) The summary of two independent biological replicates is shown. Download FIG S7, TIF file, 0.2 MB. Copyright © 2020 Herkt et al. 2020 Herkt et al. This content is distributed under the terms of the Creative Commons Attribution 4.0 International license.

**FIG 9:**
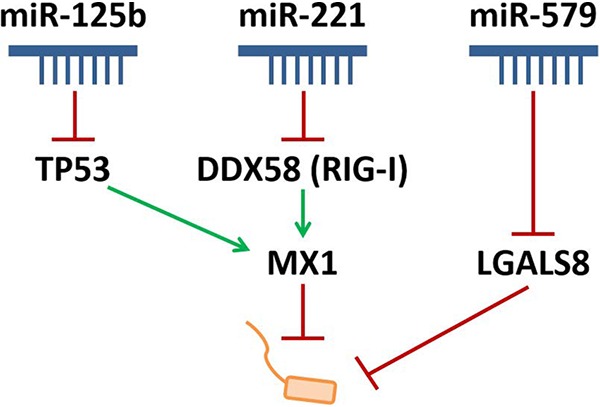
Scheme of MX1 and LGALS8 regulation by miRNA precursor pool transfection to influence L. pneumophila replication in macrophages. Precursor transfection of miR-125b reduces the expression of TP53, while DDX58 (RIG-I) is targeted by miR-221. Both targets further regulate the protein expression of MX1, which has an antibacterial effect on *Legionella*. Furthermore, miR-579 acts on LGALS8 to reduce its expression. Thus, reduction of MX1 and LGALS8 expression leads to an increased replication of *Legionella* in macrophages. In conclusion, these miRNAs are influencing the expression of their targets and have an impact on *Legionella* replication.

Second, the influence of miR-579 binding to the 3′ UTR of LGALS8 was investigated with a luciferase assay. Cotransfection of miR-579 or the miRNA-pool with the wild-type version of the 3′ UTR fragment of LGALS8 (LGALS8_WT_) revealed a significant decrease of relative luciferase activity (Fig. 8B). The transfection of miR-125b and miR-221 also led to a significantly reduced luciferase signal, but less efficiently compared to the miR-579 transfection. The transfection of the mutated version (LGALS8_mut_) with miR-579 yielded a significantly weaker decrease of relative luminescence compared to the wild-type 3′ UTR. A significant decrease was detected if miR-125b or miR-221 were cotransfected with LGALS8_mut_ compared to LGALS8_WT_. In summary, miR-579 reduced the LGALS8 3′ UTR-dependent luciferase activity, and this effect was reversed by the mutation of the miRNA seed region, illustrating a direct binding of the miR-579 to the 3′ UTR of LGALS8. In order to elaborate on the role of LGALS8 in the infection process, an siRNA knockdown of LGALS8 with subsequent CFU assay was performed. Transfected cells were infected with L. pneumophila at an MOI 0.5 for 24, 48, and 72 h. Successful LGALS8 knockdown in BDMs was verified via qPCR (Fig. 8C) and was constant over time irrespective of L. pneumophila infection. In BDMs, L. pneumophila replication was significantly increased in cells transfected with siLGALS8 (up to 150% at 72 h postinfection) (Fig. 8D). This increased L. pneumophila replication was also confirmed in differentiated THP-1 cells (Fig. S6). The major findings of this study are summarized as a schematic model in Fig. 9.

## DISCUSSION

The present study describes for the first time the global miRNA regulation in human primary macrophages in response to L. pneumophila infection. Furthermore, it identifies a new miRNA-regulated cell-autonomous immune network containing LGALS8 and the antiviral factor MX1, which restricts L. pneumophila replication in human macrophages. We found this restriction to be mediated by the repressive action of miR-125 and miR-221 on MX1 and of miR-579 on LGALS8. Downregulation of these miRNAs during infection by decreased histone H4 acetylation might therefore contribute to enhanced bacterial clearing (Fig. 9).

We identified 85 differentially expressed miRNAs in primary human macrophages infected with L. pneumophila. Exemplarily, the miRNAs miR-146a, miR-155, miR-27a, and miR-125a were upregulated in a time-dependent manner, whereas miR-221, miR-222, miR-125b, miR-26a, and miR-29b were downregulated in response to L. pneumophila infection. We have shown before that miR-146a can facilitate *Legionella* replication inside the host cell via degradation of IRAK1 mRNA (42). In the present study, miR-125b, miR-221, and miR-579 were of particular interest. While miR-579, unlike miR-125b and miR-221, was only weakly detected in our data set, we considered all three miRNAs important because of their documented role in conferring macrophage tolerance to endotoxin (31, 35) and because of their downregulation at 48 h postinfection versus control cells (see Table S1 in the supplemental material). Of note, we see a significant increase in *Legionella* CFU count upon miRNA overexpression at the same time point (Fig. 3).

Transcriptionally unrelated to miR-125b, the miR-125 family member miR-125a has been observed to be upregulated in response to infection with M. tuberculosis, L. monocytogenes (43, 44), and also L. pneumophila, as we have previously shown (45). miR-221 and miR-222 are encoded as a cluster located on chromosome X and have been reported to be overexpressed in many types of cancer (46). In addition, it has been shown that miR-221 expression was selectively decreased in lipopolysaccharide (LPS)-treated macrophages, as well as in the lungs of LPS-challenged mice (47). These observations are in line with our observation of a downregulation of miR-125b and miR-221 in response to a *Legionella* infection, and we extended our findings from BDMs to THP-1 cells. Other studies also confirmed that the regulation of miRNAs such as miR-146a/b, miR-132, miR-155, miR-214, and miR-195a, was comparable in THP-1 and primary macrophages (48), supporting our conclusion that the miRNA profile in THP-1 cells seems to be largely comparable to that of primary BDMs. Since the response of differentiated THP-1 cells to a *Legionella* infection is similar to that of primary cells with respect to key inflammatory mediators, THP-1 cells were used as cell line model instead of BDMs when necessitated by experimental upscaling.

In order to understand the mechanism of miRNA regulation in response to L. pneumophila infection, transcriptional changes of miRNAs were correlated with a genomewide histone analysis of H4 acetylation after L. pneumophila infection of human primary macrophages. Acetylation of histones is one of the master regulators of gene expression in mammalian development and human disease (49) and can strongly impact inflammatory and host defense responses (50). We have previously shown that activating acetylation upon *Legionella* infection depends on recognition of flagellin by the host (51). The correlation of selected pri-miR expression with the acetylation level of the respective promoter region following infection with L. pneumophila supported the notion that the investigated miRNAs are regulated in macrophages in response to a L. pneumophila infection by chromatin-associated events. As shown previously, *Legionella* can influence the histone code of the host by secreting effector proteins to repress gene expression (52). We therefore aimed to establish whether the regulation pattern of these miRNAs is of beneficial or deleterious consequence for the host.

It has been previously established that miR-125b, miR-221, and miR-579 are instrumental in the process of endotoxin tolerance (31, 35, 53). Reversal of the observed downregulation of this set of miRNAs postinfection by transfection caused an increased replication of L. pneumophila, whereas enhanced downregulation of all three miRNAs led to a decreased replication of L. pneumophila. A combination of all three miRNAs was necessary for this effect. Upon confirmation of a probacterial role of miR-125b, miR-221, and miR-579, we attempted to identify downstream effector molecules. In a proteomics approach, MX1 protein was found to be downregulated after transfection of all three miRNA precursors. Given its established role in restricting viral replication (54–61), it was further investigated. The human gene MX1 encodes a 78-kDa protein called MX1 or MxA (62, 63) and belongs to and shares many properties of the dynamin superfamily of large GTPases (64). MX1 proteins appear to assemble into long filamentous structures at physiological salt concentrations and are detectable by electron microscopy. In the presence of guanosine nucleotides, the filaments rearrange into rings that tubulate negatively charged liposomes and associate with the smooth ER (65, 66), hypothetically functioning in intracellular protein transport and sorting (67). The nonconstitutive expression of MX1 is tightly regulated by type I and II IFNs (68). The restrictive effect of type I IFNs on *Legionella* growth in epithelial-like cell lines and macrophages has already been documented (69–72). Although it has been previously described that interferon-related genes, including MX1, are upregulated following transfection of random 23-mer double-stranded miRNA mimics (73), we did not observe an increase of MX1 expression in the present study. The antiviral activities of human MX1 are mediated by the direct interference with, for instance, synthesis and/or nuclear import of newly synthesized viral components (74, 75). In our study, a knockdown of constitutive MX1 led to enhanced intracellular bacterial replication. It is known that L. pneumophila manipulates various key host regulatory pathways in order to establish an LCV. The LCV is decorated with many bacterial and host factors to prevent bacterial degradation and enable intracellular replication. Proteomics screens have identified several small and large GTPases that are associated with the LCV. It was demonstrated that these GTPases are functionally involved in the LCV formation, as well as in the *Legionella* replication process (76, 77). The secreted *Legionella* effector protein Lpg1137 binds and cleaves syntaxin 17 during intracellular replication, leading to an altered activity of the large GTPase Drp1 (78). We extended this observation to MX1, since siRNA- or miRNA-mediated downregulation led to increased *Legionella* replication. Because MX1 could not be found in the proteome of LCVs within U937 macrophages infected with L. pneumophila strain AA100/130b (79), we hypothesize that MX1 is not directly attached to the LCV.

While we ruled out a direct effect of miR-125b, miR-221, and miR-579 on MX1 mRNA, Ingenuity Pathway Analysis revealed DDX58 and TP53 as possible functional targets, which was confirmed by luciferase assay and knockdown experiments. The gene DDX58 encodes the cytosolic RIG-I-like receptor (RLR) RIG-I, which recognizes cytosolic double-stranded RNA or single-stranded RNA containing 5′-triphosphate. An activation of RIG-I triggers a large-scale amplification of a signaling cascade leading to the expression of type I IFN-dependent genes and induction of proinflammatory cytokines (80). In our study, DDX58 knockdown led to reduced intracellular MX1 protein. Furthermore, the targeting of DDX58 by miR-221 was demonstrated. The involvement of miR-221 in the type I IFN response has been established to occur via the RIG-I/MDA5 pathway (81), and DDX58 could be the as-yet-missing miR-221-responsive molecule limiting the viral replication by inducing the type I IFN response. It has been shown that L. pneumophila secretes the factor SdhA, which suppresses the RIG-I/MDA5 pathway (36), suggesting the importance of this pathway to influence *Legionella* replication. It is possible that the direct inhibition of RIG-I by miR-221, as well as the inhibited activation of MX1 by RIG-I, is contributing to the replication effect observed in this study.

TP53, the other established miRNA-responsive controller of MX1, is a direct target of miR-125b (37–39) and is a master regulator of the cellular mechanisms controlling responses to cellular stress such as DNA damage, aberrant oncogene activation, loss of normal cell-cell contacts, nutrient deprivation, and abnormal reactive oxygen species production. Importantly, it is also induced in response to viral infection as a downstream transcriptional target of type I IFN signaling (82). In addition, TP53 is also activated indirectly by type I IFN through other IFN-inducible proteins, such as promyelocytic leukemia protein, STAT1, or IFIXα-1 (83–85). Several studies showed that TP53 has a role in controlling bacterial infection and that inhibition of TP53 may confer certain selective advantages to bacteria (86). An inhibition of TP53 activity was observed for infection with H. pylori (87, 88), different *Mycoplasma* species (89), and Chlamydia trachomatis (90). *Chlamydia* and *Legionella* are intracellular bacteria, and they strictly rely on host resources. Since metabolic control of TP53 provides antibacterial protection, inhibition of TP53 seems to be important for bacterial survival and growth, as seen in this study. Of note, the association of TP53 and MX1 in IFN signaling has already been shown. An increased expression of TP53 is accompanied by enhanced expression of MX1, as well as RIG-I and IRF7, which strengthens its implication in the type I IFN signaling (40). In summary, counteracting the type I IFN response by DDX58 and TP53 knockdown led to an increased replication of L. pneumophila, which in our model is due to the downstream effector molecule MX1.

In an attempt to find a mechanism behind the crucial, yet unexplained role of miR-579, pathway analysis revealed the putative targets DDX58 and LGALS8 that might be effectors of MX1 regulation. Galectin-8 (LGALS8) was detected by mass spectrometry to be downregulated following transfection of all three miRNA precursors. Expression of LGALS8 was significantly reduced following miR-579 precursor transfection, and this interaction was validated by luciferase assay, establishing for the first time a targeting of LGALS8 by miR-579. Luciferase assay results suggest that more, as-yet-unidentified miR-579 binding sites might exist. Galectins are glycan-binding, evolutionary conserved proteins and have pleiotropic roles in innate and adaptive immune responses (91, 92). Galectins are localized in the cytosol and in the nucleus, and several of these galectins are also secreted (91). Within the cytosol, galectins prevent the formation of complex carbohydrates. Thus, they function as danger and pattern recognition receptors. For instance, galectin-3 accumulates on damaged bacterium-containing vesicles (93), notably including the LCV (94). Galectin-3 has been established as a marker for vacuole integrity during infection with S. enterica serovar Typhimurium, Shigella flexneri, and Trypanosoma cruzi (93, 95). It was shown that the cytosolic galectin-8 also localizes to damaged vesicles and restricts *Salmonella* replication in HeLa cells (27). In addition, as shown in our study (Fig. 8C and D), an siRNA-mediated knockdown of galectin-8 led to an increased replication of L. pneumophila. While galectin-8 was suggested to serve as a versatile receptor for vesicle damage (27), galectin-3 and galectin-8 have been demonstrated to support localization of antimicrobial guanylate binding proteins (GBPs) to bacterial vacuoles (96). GBPs, in turn, have a known role in the defense against *Legionella* (97, 98). Thus, the absence of galectin-8 upon knockdown could explain the enhanced replication of *Legionella* (27). An association of galectin-8 with the LCVs of infected human macrophages at 6 h postinfection was previously validated, strengthening the putative role as a danger receptor (99).

In summary, L. pneumophila infection induced transcription-dependent downregulation of specific miRNAs. This leads to a derepression of MX1 and more efficient controlling of L. pneumophila replication, as suggested by miRNA overexpression, which increased bacterial replication. While the troika of miR-125b, miR-221, and miR-579 has already been described in the context of tolerance to bacterial components, the mechanism we propose here, centrally involving MX1, seems to be embedded in a different functional framework.

## MATERIALS AND METHODS

### Chemicals and antibodies.

RPMI 1640, fetal calf serum (FCS), and goat anti-mouse Alexa Fluor 555 (A21428) were obtained from Life Technologies (Darmstadt, Germany). Phorbol myristate acetate (PMA) and human AB serum were purchased from Sigma-Aldrich Chemie GmbH (Taufkirchen, Germany). Paraformaldehyde was acquired from Carl Roth GmbH & Co. KG (Karlsruhe, Germany). Anti-MX1 antibody (ab95926) was obtained from Abcam, Plc (Cambridge, United Kingdom), while horseradish peroxidase (HRP)-linked anti-rabbit antibody (5127S) was acquired from Cell Signaling Technology (Leiden, Netherlands). Anti-tubulin (sc-5286), HRP-linked anti-mouse antibody (sc-2005), and anti-rabbit IgG control (sc-66931) were purchased from Santa Cruz Biotechnology, Inc. (Santa Cruz, CA). All chemicals used were analytical grade and obtained from commercial sources.

### *L. pneumophila* culture.

L. pneumophila strain Corby wild type or a *gfp*-expressing strain (kindly provided by K. Heuner, RKI, Berlin, Germany) was routinely grown on buffered charcoal-yeast extract (BCYE) agar plates at 37°C for 3 days. Infection was carried out as described before (42).

### Cell culture and *L. pneumophila* infection.

THP-1 cells (ATCC) and primary monocytes were cultured and differentiated as previously described (32). For all infection experiments, differentiated THP-1 cells or BDMs were infected with L. pneumophila strain Corby at an MOI of 0.25 or 0.5 or left uninfected as control. For SILAC (stable isotope labeling of amino acids in cell culture) experiments (100), THP-1 cells were cultivated in SILAC-DMEM with 2% (vol/vol) FCS lacking arginine and lysine. The medium was additionally supplemented with heavy-isotope-labeled [^13^C]arginine and [^13^C]lysine (Cambridge Isotope Laboratories, Tewksbury, MA), and THP-1 cells were cultured over five passages to reach a labeling efficiency of >97% for all proteins.

### Transfection of macrophages.

Lipofection of THP-1 cells or BDMs with ON-TARGET*plus* siRNA pools (Dharmacon, Lafayette, CO), targeting either MX1, DDX58, TP53, or LGALS8 mRNA was carried out according to the manufacturer’s protocol (Lipofectamine 2000; Thermo Fisher Scientific, Waltham, MA). Transfection of THP-1 cells with miRNA mimics or inhibitors was performed with siPORT NeoFX (Invitrogen, Carlsbad, CA) according to the manufacturer’s instructions. mirVana mimics and inhibitors and corresponding controls were purchased from Thermo Fisher Scientific.

### CFU assay.

To analyze bacterial replication in transfected THP-1 cells or BDMs, cells were infected with L. pneumophila at an MOI of 0.5. Cells were lysed at indicated time points with 1% saponin, and diluted lysates were streaked onto BCYE agar plates. After 3 days of incubation at 37°C, L. pneumophila colonies were counted, and the bacterial load was calculated.

### miRNA promoter prediction and differential promoter acetylation.

The putative active transcription start sites (TSS) of miR-155, miR-146a, miR-221, and miR-125b in macrophages, and their genomic locations were determined using PROmiRNA software (101). The software uses a machine learning approach to predict the TSS of primary miRNA transcripts (pri-miRNAs) based on the signal from CAGE data from the FANTOM4 project libraries (102) and sequence features such as GC content and TATA box elements. We used epigenetic data, in particular H4Ac from our previous studies (32) to identify significant acetylation changes at miRNA promoters upon infection.

### RNA preparation and real-time PCR.

For analysis of gene expression, total cellular RNA was isolated by phenol-chloroform extraction with TRI Reagent (Sigma-Aldrich). For detection of pri-miRNAs, purified RNA was digested with DNase I (Roche, Mannheim, Germany). After reverse transcription (high-capacity RNA-to-cDNA kit or TaqMan miRNA reverse transcription kit; Thermo Fisher Scientific), quantitative real-time PCR was performed on a ViiA7 (Thermo Fisher Scientific) with Fast SYBR green (Thermo Fisher Scientific) and specific primer pairs. miRNA expression was analyzed with TaqMan assays and TaqMan MasterMix to detect the mature and primary forms of the miRNAs (Thermo Fisher Scientific). The specific primers for SYBR green qPCR were hDDX58 (sense, 5′-ATCCCAGTGTATGAACAGCAG-3′; antisense, 5′-GCCTGTAACTCTATACCCATGTC-3′), hLGALS8 (sense, 5′-TTGAGATCGTGATTATGGTGCT-3′; antisense, 5′-ATCCTGTGGCCATAGAGCAG-3′), hMX1 (sense, 5′-AAGAGCTCCGTGTTGGAGG-3′; antisense, 5′-TGGTAACTGACCTTGCCTCTC-3′), hRPS18 (sense, 5′-GCGGCGGAAAATAGCCTTTG-3′; antisense, 5′-GATCACACGTTCCACCTCATC-3′), and hTP53 (sense, 5′-GGGCTTCTTGCATTCTGG-3′; antisense, 5′-CCTCCGTCATGTGCTGTG-3′).

### Illumina small RNA sequencing and bioinformatics analysis.

The miRNome of L. pneumophila*-*infected BDMs was determined by Illumina TruSeq small RNA sequencing (Illumina, San Diego, CA). BDMs of three different donors were infected with L. pneumophila at an MOI of 0.25 for 24 and 48 h or left untreated as control. miRNAs with an absolute log_2_-fold change of ≥0.65 (corresponding to a linear fold change of 1.5) and an adjusted *P* value of ≤0.2 (Benjamini-Hochberg correction) in at least one comparison were considered significantly regulated.

### SILAC.

Quantitative proteome analysis of infected THP-1 cells was performed as described previously (103). Details of this analysis are described in the supplemental information (Table S2).

10.1128/mBio.03155-19.10 TABLE S2 Parameters for LC-MS/MS analysis. Detailed information and the settings used for the reversed-phase liquid chromatography (RPLC) and mass spectrometry (MS) are indicated. Download Table S2, DOCX file, 0.03 MB. Copyright © 2020 Herkt et al. 2020 Herkt et al. This content is distributed under the terms of the Creative Commons Attribution 4.0 International license.

### ELISA.

All secreted cytokines (interleukin 1β [IL-1β], IL-6, IL-10, tumor necrosis factor alpha [TNF-α], and granulocyte-macrophage colony-stimulating factor [GM-CSF]) were measured using a Milliplex MAP human high-sensitivity T cell panel premixed 13-Plex–immunology multiplex assay (Merck Millipore) in a Bio-Plex Magpix (Luminex Corporation, Austin, TX) according to the manufacturer’s instructions.

### Western blot analysis.

Cellular proteins were harvested by lysis and protein concentration was determined by BCA assay (Thermo Fisher Scientific). For protein separation, 10% SDS gels were used with 20 to 30 μg of protein per lane. Proteins were transferred to a nitrocellulose membrane with the use of a tank blot for 1 h at 100 V. Primary antibody (α-MX1 or α-tubulin-antibody) was added at a 1:1,000 dilution, followed by incubation for 2 h at room temperature on a tumbling shaker. HRP-conjugated secondary antibody (1:2,000) was added for additional 2 h at room temperature on a tumbling shaker. After removal of excess antibody by washing, protein signal was detected on a bioluminescence and chemiluminescence imager (INTAS Science Imaging Instruments GmbH, Göttingen, Germany). When required, quantification of signal was performed by densitometric analysis, using the LabImage 1D software (Kapelan Bio-Imaging GmbH, Germany).

### Flow cytometry.

After consecutive transfection, differentiation, and L. pneumophila infection of THP-1 cells, the cells were detached with trypsin-EDTA, fixed with 4% paraformaldehyde (PFA), and permeabilized with 0.5% Tween 20, followed by blocking with 0.5% (vol/vol) Tween with 10% FCS and Human BD Fc Block (BD Bioscience, San Jose, CA). Primary α-MX1 antibody, α-LGALS8 antibody and α-IgG isotype control antibody were added for 1 h. Then, secondary anti-rabbit Alexa 555-labeled antibody was added for 30 min at ambient temperature in the dark. After a washing step, the cells were analyzed on a Guava easyCyte flow cytometer (Merck Millipore). Data were analyzed using FlowJo version 7.6.5. In order to account for signal arising from unspecific antibody binding, only signal above the IgG control intensity was considered.

### Luciferase assays.

Luciferase measurements were carried out as described previously (45). Luciferase reporter plasmids containing partial wild-type 3′ UTRs of DDX58 (NM_014314.4, 3′ UTR positions 1 to 813), TP53 (NM_001276760.2, 3′ UTR positions 701 to 1203), and LGALS8 (NM_006499.4, 3′ UTR positions 2043 to 3662) were constructed by NotI/XhoI restriction digest on the psiCHECK2 vector backbone (Promega, Madison, WI). Mutated sequences were purchased as gBlocks from IDT Technologies (Coralville, IA) and are depicted in Fig. S5 in the supplemental material. Detailed transfection methods are given in the supplemental material (Text S1).

10.1128/mBio.03155-19.1 TEXT S1 The supplemental methods contain supplementary information on methodology and corresponding literature citations. Download Text S1, DOCX file, 0.04 MB. Copyright © 2020 Herkt et al. 2020 Herkt et al. This content is distributed under the terms of the Creative Commons Attribution 4.0 International license.

### *In silico* pathway analyses.

The IPA tool (104) was used to investigate the connection between the miRNA pool (miR-125b, miR-221, and miR-579) and the MX1 protein. Detailed methods are given in the supplemental material (Text S1).

### Ethical statement.

All blood donors gave informed written consent for use of their blood samples for scientific purposes.

### Statistics.

Statistic evaluation was carried out with GraphPad Prism (version 7). For data with multiple variables, two-way analysis of variance (ANOVA) was performed, followed by either Sidak’s or Tukey’s comparison test. For comparison of two data columns, a paired *t* test was used on log_2_-transformed data. For all tests, a Gaussian distribution was assumed, and the confidence interval was set to 95%. *P* values of ≤0.05 were considered significant. All statistical tests are specified in the respective figure legends.
