# Supplementary figures and images for: A MicroRNA Network Controls Legionella pneumophila Replication in Human Macrophages via LGALS8 and MX1
Source: mBio. 2020 Mar 24;11(2):e03155-19. doi: 10.1128/mBio.03155-19 (PMC7157531; doi:10.1128/mBio.03155-19)

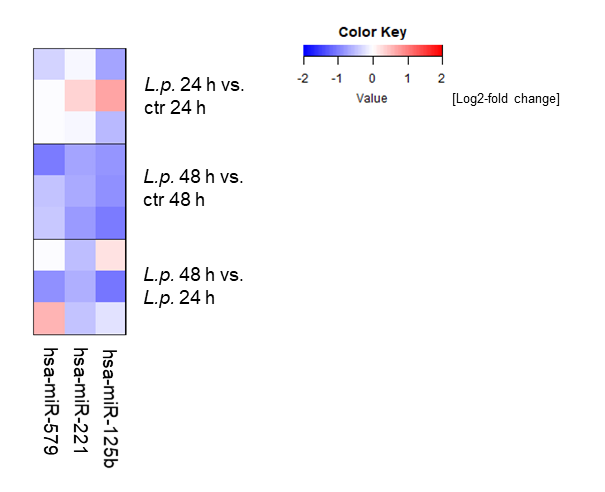

Supplement: FIG S1 [file mBio.03155-19-sf001.tif]

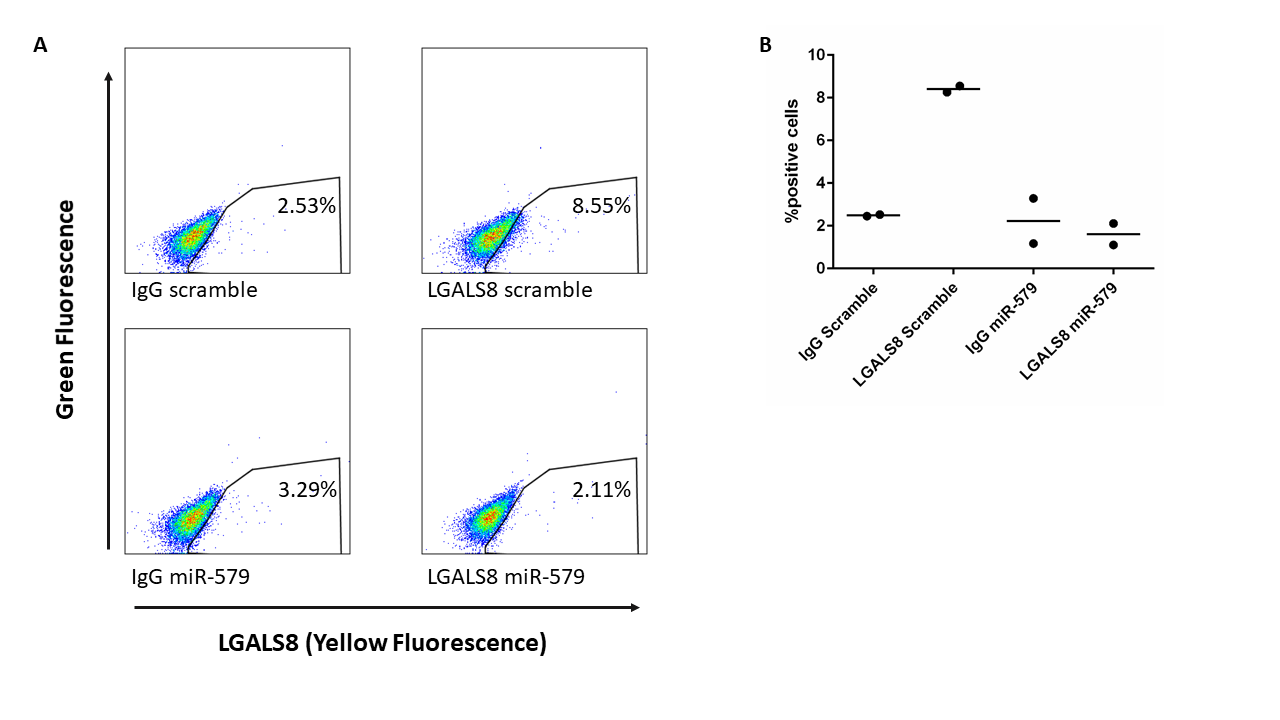

Supplement: FIG S7 [file mBio.03155-19-sf007.tif]
