# Supplementary material for: A MicroRNA Network Controls Legionella pneumophila Replication in Human Macrophages via LGALS8 and MX1
Source: mBio. 2020 Mar 24;11(2):e03155-19. doi: 10.1128/mBio.03155-19 (PMC7157531; doi:10.1128/mBio.03155-19)
Supplement: TABLE S2 [file mBio.03155-19-st002.docx]

Supplementary Information for

A miRNA network controls Legionella pneumophila replication in human macrophages via LGALS8 and MX1

Christina E. Herkt, Brian E. Caffrey, Kristin Surmann, Sascha Blankenburg, Manuela Gesell Salazar, Anna Lena Jung, Stefanie M. Herbel, Kerstin Hoffmann, Leon N. Schulte, Wei Chen, Alexandra Sittka-Stark, Uwe Völker, Martin Vingron, Annalisa Marsico, Wilhelm Bertrams, Bernd Schmeck

Bernd Schmeck

Email: [bernd.schmeck@uni-marburg.de](mailto:bernd.schmeck@uni-marburg.de)

**This PDF file includes:**

Table S2

Table S2. Parameters for LC-MS/MS analysis. Detailed information and used settings of the reversed phase liquid chromatography (RPLC) and the mass spectrometry (MS).

| **reversed phase liquid chromatography (RPLC)** | |
| --- | --- |
| instrument | Ultimate 3000 RSLC (Thermo Fisher Scientific) |
| trap column | 75 μm inner diameter, packed with 3 μm C18 particles (Acclaim PepMap100, Thermo Fisher Scientific) |
| analytical column | Accucore 150-C18, (Thermo Fisher Scientific)  25 cm x 75 μm, 2,6 μm C18 particles, 150 Å pore size |
| buffer system | binary buffer system consisting of 0.1% acetic acid, 2% ACN (buffer A) and 100% ACN in 0.1% acetic acid (buffer B) |
| flow rate | 300 nl/min |
| gradient | linear gradient of buffer B from 2% up to 25% |
| gradient duration | 60 min |
| column oven temperature | 40°C |
| **mass spectrometry (MS)** | |
| instrument | Q Exactive mass spectrometer (Thermo Fisher Scientific) |
| operation mode | data-dependent |
| **Full MS** |  |
| MS scan resolution | 70,000 |
| AGC target | 3e6 |
| maximum ion injection time for the MS scan | 120 ms |
| Scan range | 300 to 1650 m/z |
| Spectra data type | profile |
| **dd-MS2** |  |
| Resolution | 17,500 |
| MS/MS AGC target | 2e5 |
| maximum ion injection time for the MS/MS scans | 120 ms |
| Spectra data type | centroid |
| selection for MS/MS | 10 most abundant isotope patterns with charge ≥2 from the survey scan |
| isolation window | 3 *m/z* |
| Fixed first mass | 100 m/z |
| dissociation mode | higher energy collisional dissociation (HCD) |
| normalized collision energy | 27.5% |
| dynamic exclusion | 30 s |
| Charge exclusion | 1>6 |
