# Supplementary material for: A MicroRNA Network Controls Legionella pneumophila Replication in Human Macrophages via LGALS8 and MX1
Source: mBio. 2020 Mar 24;11(2):e03155-19. doi: 10.1128/mBio.03155-19 (PMC7157531; doi:10.1128/mBio.03155-19)
Supplement: TEXT S1 [file mBio.03155-19-s0001.docx]

Supplementary Information for

A miRNA network controls Legionella pneumophila replication in human macrophages via LGALS8 and MX1

Christina E. Herkt, Brian E. Caffrey, Kristin Surmann, Sascha Blankenburg, Manuela Gesell Salazar, Anna Lena Jung, Stefanie M. Herbel, Kerstin Hoffmann, Leon N. Schulte, Wei Chen, Alexandra Sittka-Stark, Uwe Völker, Martin Vingron, Annalisa Marsico, Wilhelm Bertrams, Bernd Schmeck

Bernd Schmeck

Email: [bernd.schmeck@uni-marburg.de](mailto:bernd.schmeck@uni-marburg.de)

**This file includes:**

Supplementary Methods

References for SI reference citations

Supplementary Information Text

Methods

**Cell culture and *L. pneumophila* infection**

THP‑1 cells (ATCC) and were differentiated into a macrophage-like phenotype using 20 nM PMA for 24 h and then plated at desired density. Primary monocytes were isolated by MACS CD14 positive selection from donor buffy coats provided by the Centre for Transfusion Medicine and Haemotherapy in Giessen, Germany. Freshly isolated monocytes were seeded in ultra-low attachment plates (Corning, Sigma-Aldrich Chemie GmbH, Corning, USA). Cells were left to adhere for two hours in RPMI medium without supplements. Afterwards, adhesion of cells was validated by microscopy, and 1% (v/v) of human AB serum was added. Monocytes were cultivated for 6 days. Maturation to BDMs was confirmed by microscopy.

The medium was additionally supplemented with heavy isotope labeled (13C) arginine and lysine (Cambridge Isotope Laboratories, MA, USA) and THP-1 cells were cultured over five passages to reach a labeling efficiency of >97% for all proteins. In order to ensure high coverage of proteins in the subsequent internalization experiment, parts of the heavily labeled THP-1 cells were additionally infected with L. pneumophila. 13C labeled cells were harvested in aqueous buffer containing 8 mM urea and 2 mM thiourea and stored at -80°C in aliquots until usage.

**miRNA promoter prediction and differential promoter acetylation**

miRNA promoter regions were defined as +/- 1000 base pairs around the predicted transcription start site. The input-normalized promoter read counts from the H4Ac ChIP sequencing experiment, as well as the promoter fold change enrichment/depletion in infected versus non-infected samples, were computed as in ([1](#_ENREF_1)).

**Illumina small RNA sequencing and bioinformatics analysis**

RNA quality was examined on a Bioanalyzer 2100 (Agilent Technologies, Santa Clara, USA). Samples with sufficient RNA quality (RIN > 8) were used for Illumina library preparation according to the manufacturer´s instructions (Illumina). Reads were processed with the ‘Mapper’ module of miRDeep2 ([2](#_ENREF_2)). Raw read counts for both the mature and the star strand of each miRNA were determined with the ‘Quantifier’ module of miRDeep2, based on miRbase annotation v20 ([3](#_ENREF_3)). miRNA raw read counts for all 12 libraries were quantile-normalized and differential expression analysis between all pairwise conditions was performed with the Limma R package.

Briefly, Solexa output files were converted to fasta files, 3’ adapters were clipped from the reads, reads shorter than 18 nucleotides (nt) were discarded and reads with identical sequence were collapsed to remove redundancy. Reads were mapped to the human genome, hg19 assembly, using bowtie v1 and only alignments with zero mismatches in the first 18 nt of the read were kept. Up to two mismatches were allowed after 18 nt. Only reads which aligned to less than five multiple positions were kept for further analysis.

**SILAC**

In brief, THP‑1 cells (from infection experiments and the SILAC standard) were disrupted and the protein content of each sample was determined using a Bradford assay (Biorad, Munich, Germany). Samples from the infection experiment were mixed with equal protein amounts of the SILAC standard ([4](#_ENREF_4)). The resulting combined samples were separated using one-dimensional gel (1D gel) electrophoresis with NuPAGE4-12% acrylamide Bis-Tris Midi Gels (Novex Life Technologies, Darmstadt, Germany) according to manufacturer’s instructions. Each sample lane on the 1D gel was cut into ten equally sized slices. Proteins were digested with trypsin and extracted from the gels as described before ([5](#_ENREF_5)). ACN was removed by lyophilisation, samples were reconstituted in 40 µl 1% (v/v) acetic acid prior to purification using C18 ZipTip columns (Merck Millipore, Burlington, USA). After one more lyophilisation step, peptides were dissolved in 20 µl buffer A (2% ACN, 0.1% acetic acid in HPLCgrade water (Baker, USA) before nanoHLPC-MS/MS. Peptides were analyzed on a Q Exactive mass spectrometer (Thermo Fisher Scientific) in data dependent mode after separation on a Dionex UltiMate 3000 nanoLC system (Dionex/Thermo Fisher Scientific, Idstein, Germany) and ionization with a TriVersa NanoMate source (Advion, Ltd., Harlow, UK). Details of the LC-MS/MS analysis are provided as Supporting Information Table S1. MS data were searched using MaxQuant ([6](#_ENREF_6)) against a database derived from Uniprot limited to human entries (version 06-2015). Oxidation at methionine and 13C labeling (+6.02 Da) at arginine and lysine were set as dynamic modifications, carbamidomethylation of cysteine was set as fixed modification and no missed cleavage of trypsin was allowed. The false discovery rate of peptide identifications was set to < 1%. Only proteins which were detected with one or more than one peptide, if they contributed to at least 10% of the protein sequence coverage, were considered for further analysis. Furthermore, proteins had to be detected in at least three of the four biological replicates per condition for calculation of protein ratios between the conditions. From the MaxQuant analysis, normalized SILAC ratios (light to heavy) of the peak areas in the experimental samples against the global SILAC standard were retrieved on protein level. These data were compared between infection and control depending on the presence of miRNA and the point in time of harvest. P- and q-values (Benjamini-Hochberg correction) were calculated using the Genedata Analyst v8.0 software (Genedata AG, Basel, Switzerland). Proteins were defined as regulated if the absolute fold change exceeded 1.2 and the p-value was < 0.05. Venn diagrams were created using Venny 2.0 ([7](#_ENREF_7)).

**Luciferase Assay:**

HEK‑293T cells were transfected with Lipofectamine 2000 at a final concentration of 2.5 pmol per miRNA mimic and 200 ng reporter plasmid. Specific miRNA mimics for miR‑125b, miR‑221 and miR‑579 were used. As control, a scrambled mimic sequence was transfected. Parallel transfection of three miRNA mimics (miRNA-pool) was performed at equimolar concentrations (0.833 pmol per miRNA mimic, totaling at 2.5 pmol). The bare psiCHECK2 vector was transfected in combination with the miRNA mimics to account for non-specific miRNA/vector interaction.

## In silico pathway analyses

In Ingenuity Pathway screening, filters were set to only include experimentally observed or high-confidence predicted miRNA/mRNA interaction partners. Output was limited to the most stringent candidates. The newly found miRNA targets were interconnected using all available data sources in IPA with restriction to experimentally validated or high-confidence predicted interactions.

**References**

1. Du Bois I*, et al.* (2016) Genome-wide Chromatin Profiling of Legionella pneumophila-Infected Human Macrophages Reveals Activation of the Probacterial Host Factor TNFAIP2. *The Journal of infectious diseases* 214(3):454-463.

2. Friedlander MR, Mackowiak SD, Li N, Chen W, & Rajewsky N (2012) miRDeep2 accurately identifies known and hundreds of novel microRNA genes in seven animal clades. *Nucleic acids research* 40(1):37-52.

3. Kozomara A & Griffiths-Jones S (2014) miRBase: annotating high confidence microRNAs using deep sequencing data. *Nucleic acids research* 42(D1):D68-D73.

4. Surmann K*, et al.* (2015) A proteomic perspective of the interplay of Staphylococcus aureus and human alveolar epithelial cells during infection. *J Proteomics* 128:203-217.

5. Samal R*, et al.* (2012) OMICS-based exploration of the molecular phenotype of resident cardiac progenitor cells from adult murine heart. *Journal of proteomics* 75(17):5304-5315.

6. Cox J & Mann M (2008) MaxQuant enables high peptide identification rates, individualized p.p.b.-range mass accuracies and proteome-wide protein quantification. *Nature biotechnology* 26(12):1367-1372.

7. Oliveros JC (2007-2015) Venny. An interactive tool for comparing lists with Venn's diagrams. <http://bioinfogp.cnb.csic.es/tools/venny/index.html>.
